# Current Status of HIV-1 Vaccines

**DOI:** 10.3390/vaccines9091026

**Published:** 2021-09-16

**Authors:** Anna Hargrave, Abu Salim Mustafa, Asma Hanif, Javed H. Tunio, Shumaila Nida M. Hanif

**Affiliations:** 1 Department of Biomedical Sciences, Kentucky College of Osteopathic Medicine, University of Pikeville, Pikeville, KY 41501, USA; annahargrave@upike.edu; 2 Department of Microbiology, College of Medicine, Kuwait University, Kuwait City 12037, Kuwait; abu.mustafa@ku.edu.kw; 3 Department of Restorative Sciences, College of Dentistry, Kuwait University, Kuwait City 12037, Kuwait; Asma.h@hsc.edu.kw; 4 Department of Internal Medicine, Carver College of Medicine, University of Iowa, Iowa City, IA 52242, USA; Javed-tunio@uiowa.edu

**Keywords:** HIV, vaccines, clinical phases

## Abstract

HIV-1 infection and its progression to AIDS remains a significant global health challenge, particularly for low-income countries. Developing a vaccine to prevent HIV-1 infections has proven to be immensely challenging with complex biological acquisition and infection, unforeseen clinical trial disappointments, and funding issues. This paper discusses important landmarks of progress in HIV-1 vaccine development, various vaccine strategies, and clinical trials.

## 1. Introduction

In 2020, 37.6 million people were living with HIV infections, and 1.5 million people acquired HIV infections within the year. Since the HIV epidemic began, 34.7 million people have died due to an AIDS-related illness. In 2020, 690,000 people died due to an AIDS-related illness [1]. It has been estimated that 85% of all HIV cases are transmitted sexually. In contrast, the other 15% of cases are transmitted from shared injection needles, infected blood transfusions, or from mother to child [2].

Treatment of HIV-1 infections drastically changed as antiretroviral drugs evolved. This started with azidothymidine, an inhibitor of viral reverse transcriptase, in 1987, and it decreased the amount of HIV RNA in the bloodstream. This treatment plan was altered from a single-drug regimen for a more effective two-drug regimen. Then, clinicians tested a three-drug regimen, including the newly developed protease inhibitors, and this was referred to as the highly effective combination antiretroviral therapy (ART) in 1996. Provided that patients with HIV-1 infections consistently adhere to ART, they have close to normal life expectancies and do not transmit the virus to an uninfected sexual partner. The virus is not transmitted in this circumstance because ART suppresses the level of the virus to incredibly low levels [3].

Fourteen years later, in 2012, clinicians started using ART to prevent HIV-1 infections and referred to this as preexposure prophylaxis or PrEP. A single pill taken once daily has been shown to be 99% effective in preventing HIV-1 infection via sexual acquisition [3]. Currently, all people at high risk for HIV infection should be offered PrEP according to the US Preventive Services Task Force Grade A [3].

While the development of effective antiretroviral drugs for patients with HIV-1 infections and their application as PrEP to help prevent infection to at-risk people is an important landmark in scientific history, it does not replace the need for an effective vaccine [3,4,5,6]. Given that developing countries have approximately 90% of the people with HIV-1 infections and antiretroviral drugs are inaccessible, it is clear that a vaccine is required to end this epidemic [4].

To end this epidemic, two possible immunization strategies must be considered as possible solutions, therapeutic and prophylactic vaccines. The aim of prophylactic vaccines is to prevent the infection or disease while therapeutic vaccines are aimed at treating the individual already infected with HIV. This review is focused on the prophylactic vaccines, and as such, it considers clinical trials that assess the risk of contracting HIV-1 infections after receiving a prophylactic vaccine instead of reduction in viral load.

## 2. Challenges of the HIV-1 Vaccine

### 2.1. Biological Perspective

The biological challenges of HIV vaccine development include a high rate of mutation and recombination during viral replication, four main groups of HIV with nine subtypes/clades across the world, no appropriate animal models, and limited information regarding the correlates of immune protection.

The high rate of mutation of HIV is due to the error-prone viral reverse transcriptase and has been estimated to lead to 1–10 mutations per genome per replication cycle. This mutation rate mostly leads to changes within the Env glycoprotein, allowing glycan shielding so the virus can evade the immune system. Though there is considerable genetic diversity present in the Env glycoprotein, this structure is the main target of neutralizing antibodies [6].

HIV-1 infections are classified into a group (M, N, O, P) and subtype or clade (A, B, C, D, F, G, H, J, K). Within a clade, genetic variation can be as high as 30%. Additionally, 10–20% of people infected with HIV in certain regions of Africa have two or more viral subtypes [6]. These viral subtypes lead to recombinant strains such as A/Ga recombinant in West Africa and B/C recombinant in China [2].

The lack of appropriate animal models posed a unique challenge to researchers before the early 1990s. Chimpanzees, an endangered species classified as a nonhuman primate (NHP), contracted HIV, but it did not follow the course of the disease in humans. This led to the US and Japan separately developing SHIV, a chimeric virus with gag and pol genes from SIV and env gene from HIV, because it is a more pathogenically relevant model for the generation of an HIV vaccine. The standard animal model widely accepted today is macaque monkeys infected with SHIV administered with low doses and intravaginally [7]. It is worth noting that this standard accepted animal model is only a model and may not reflect the disease pathology and immune response in humans.

Researchers are also challenged by the lack of information regarding the correlates of immune protection. This is due to the complex progression of HIV-1 infection since the infection is never able to be cleared by the immune system because of the reservoir of latently infected memory CD4+ T-cells [4]. During the preliminary stage of infection for the majority of patients, T-cells attack immunodominant highly variable regions of HIV-1, leading to escaped HIV-1 variants with decoy epitopes and ineffective protection against them [8].

An interesting characteristic that may provide insight into immune correlates is the three unique subsets of the population with HIV-1 infections. There are viremic controllers who suppress the viral load from 50 to 2000 RNA copies per mL, elite controllers who suppress the viral load to <50 RNA copies per mL, and long-term nonprogressors. Long-term nonprogressors maintain stable CD4+ T-cells above 500 cells per microliter for a decade without ART. The mechanisms behind these subsets’ response to HIV-1 are not fully understood. Some genetic studies suggest the difference in amino acids in proteins that code for HLA class I alleles are correlated to controllers or progressors. HLA-B*57, HLA-B*27, HLA-B*52, and HLA-B*14 are more frequently found in controllers, while HLA-B*07, HLA-B*08, and HLA-B*25 are linked to an increased risk of progression. Though genetic variation is an important aspect of understanding the biology behind viremic and elite controllers, no HLA Class I allele is able to suppress the virus. Researchers believe that the mechanism likely involves HLA association with CD8+ T-cells and other nongenetic factors [9]. 

Another characteristic that may provide insight into immune correlates is the neutralizing antibodies present in approximately 10–30% of people with HIV-1 infections. These antibodies are developed from heavy chain mutation and are not produced until years into a chronic natural infection [10]. While they do not provide any protection, they may offer information regarding what is needed for the immune system to prevent HIV-1 acquisition.

### 2.2. Funding

There is a gap between the resources and funding needed to develop an effective vaccine and the countries that have large populations with HIV-1 infections. Unfortunately, the financial backing behind HIV vaccine research and development has decreased since 2010. Eighty-five percent of the funding has been contributed by the US government and the Bill & Melinda Gates Foundation. In 2018, the combined contribution from these two groups amounted to approximately 680 million US dollars [11].

### 2.3. A Brief History of HIV Vaccine Development

From 1987 to 2021, there have been three major approaches driving HIV-1 vaccine development. Each of these approaches involved one or more clinical trials and is summarized in Table 1. Though the first two approaches have mostly been concluded, it is worth noting that each approach has been continually reexplored as researchers learn more [7,12].

In the late 1980s, the first approach focused on generating a vaccine that would induce neutralizing antibodies because neutralizing antibodies and their associated subsequent cytotoxic T lymphocyte responses were believed to provide enough protection against HIV-1. Vaccines designed and tested targeted gp120 or gp160 HIV-1 envelope proteins [13]. This was based on the observation that neutralizing antibodies could be produced in response to envelope glycoproteins present on the virus because this had occurred with the recombinant hepatitis B vaccine. This approach mostly ended in 2003, after the VaxGen trials testing gp120 vaccines produced poor results [12].

The second approach was based on administering a viral vector to induce a CD8+ T cell response [13]. In the early 2000s, researchers started focusing on how CD8+ T-cells controlled HIV infection [12] because when CD8+ T-cells decreased significantly during acute infection, the immune system was no longer able to control the virus, as observed in animal and human studies [6]. The goal in inducing a CD8+ T cell reaction was to control post-infection viremia and potentially prevent HIV acquisition. Using a recombinant vector with HIV genes as a vaccine, the virus would produce HIV proteins that would be presented to the immune system via the Class I antigen-presenting pathway [14]. This approach ended approximately after the STEP trial was terminated [12].

Briefly, the STEP trial starting in 2004 and its associated counterpart, “Phambili”, starting in 2007 were the first T-cell-based vaccine candidates. Both tested recombinant Ad5 vector with HIV-1 clade B gag/pol/nef inserts. It is worth noting that no envelope genes were present [14] because this would allow the immune system to attack the proteins and DNA within the core of the virus. Both clinical trials were ended prematurely because the STEP trial provided no efficacy and did not decrease viral load in participants who contracted HIV. The results also indicated that some participants who had Ad5-neutralizing antibodies and/or were uncircumcised were more likely to contract HIV as compared to the placebo group [7,14,15]. Hence, it was an example of product failure.

The third and current approach is to utilize a heterologous prime-boost to elicit humoral and cell-mediated immune responses [13]. The prime-boost strategy is based on priming with a virus and boosting with a recombinant protein. A homologous prime-boost is utilized for diphtheria, tetanus, and pertussis (DTP) and involves administering the same vaccine at intervals to boost the previous responses. The heterologous prime-boost utilizes the same antigens in different types of vaccines and has been proven to be more immunogenic than the homologous series [16]. This has been employed in numerous clinical trials, and it has been able to significantly improve the humoral and cellular immune response while simultaneously inducing neutralizing antibodies [6]. Prime-boost strategies’ outcome change is based on the selection of antigen, vector type, delivery route, dose, adjuvant, schedule, and sequence of immunizations [17]. Theoretically, this approach provides a heightened immune response (in terms of breadth and depth) that is focused on the inserts, not the vectors, and produces unique populations of effector-like memory T-cells that gather at the nonlymphoid organs [17].

This approach was employed in the only modestly successful trial to date, RV 144, and its results were made public in 2009, only two years after STEP’s disappointing results [7]. This trial is discussed in detail under “Milestone Event”.

Though the prime-boost approach has proven to be somewhat successful, there has yet to be an HIV vaccine, so more research into the understanding of HIV’s mechanism and its immune correlates, development of an effective and safe vaccine, and clinical trials are necessary.

**Table 1:** Historic phase 2b and 3 HIV-1 vaccine clinical trials.

Name	Year Started and Country/Continent	Phase	Molecular Basis of Vaccine	Efficacy/Response	Relevant Information	NCT Number or Author
Vax 003	1999, Thailand	3	AIDSVAX B/E	No efficacy	Bivalent subunit vaccine, 2 Gp120 from clades B and E were combined and alum adjuvant added	NCT00006327
Vax 004	1998, North America and The Netherlands	3	AIDSVAX B/B	No efficacy	Bivalent subunit vaccine, 2 Gp120 from clade B were combined and alum adjuvant added	NCT00002441
RV 144	2003, Thailand	3	ALVAC-HIV and AIDSVAX B/E	31.2% efficacy against HIV-1 acquisition	NA	NCT00223080
HVTN 502/Step and HVTN 503/Phambili	2004, North and South America, Australia, Caribbean, and South Africa	2b	MRKAd5 HIV-1 gag/pol/nef B	No efficacy	Both studies prematurely terminated. People with high titer to adenovirus were more likely to contract HIV. Uncircumcised men had a higher risk of contracting HIV [7,14,15].	NCT00095576 and NCT00413725

### 2.4. Important Advances in Scientific Technology Impacting HIV Vaccine Development

Since researchers have been developing an HIV vaccine for two and a half decades, the fields of molecular biology, bioinformatics, and “omics” based technology have all developed from inception or significantly expanded [2,12].

Molecular biology and bioinformatics techniques rapidly evolved and led to the HIV genome sequencing and cloning and identification of structural proteins of the virus [12]. “Omics” based technology ranging from “vaccinomics,” genomics, and reverse immunology allowed researchers to design highly specific DNA vaccines [2].

Since the beginning of HIV vaccine development, it is worth noting the significant changes from the initial recombinant vectors to more effective and safer vectors. This transition to improve recombinant vectors was specifically between the first and second approaches discussed in the history of HIV development section. Initially, a recombinant vaccinia virus was utilized in 1987, and it posed serious potential concerns [17]. Individuals who had already received the smallpox vaccine would not mount an appropriate immune response if vaccinated against HIV-1 in the same vector, so receiving this vaccine would be a futile effort. Immunocompromised individuals likely would be severely ill because of the replicating virus [17].

In 1975, researchers developed MVA, a nonreplicating highly attenuated vaccinia virus with over 200 poxvirus proteins [18]. It was tested and proven to be safe, inducing a cell-mediated immune response [17]. In the early 1990s, scientists developed two other nonreplicating poxviruses, NYVAC (highly attenuated vaccinia virus) and ALVAC (an avian poxvirus, canarypox). Both of these poxviruses are considered to be safe [6]. Currently, the most common recombinant vectors utilized in clinical trials since 2015 are ALVAC, Ad26, and/or MVA, as given in Table 2. Though recombinant vector is an obvious aspect of immunization, it determines immunogenicity and can drastically change the clinical trial results. An example of this is that plasmid vectors containing Env or Gag in the full-length form have poor immunogenicity and are ineffective. To circumvent this, several clinical trials rely on administering the plasmid with the HIV gene followed by a highly immunogenic recombinant vector [2].

### 2.5. Milestone Event

Given the numerous challenges in HIV-1 vaccine development, scientists doubted whether a vaccine could be generated to provide immunity against this virus. One significant breakthrough that illustrated that a preventative HIV-1 vaccine is possible was the result of the RV144 trial, obtained in 2009 [14].

Briefly, a summary of the RV144 trial protocol is as follows. This phase 3 efficacy trial conducted in Thailand tested ALVAC-HIV, a recombinant canarypox vector. This vector included Env (clade E), group-specific antigen (gag) (clade B), and protease (pro) (clade B) and is classified as a prime [6]. Following the priming events, study participants also received AIDSVAX, a protein boost with alum as an adjuvant. This protein boost was a combination of gp120 clade B (Note that this protein was modified. Eleven amino acids from N-terminal were deleted, and protein was tagged with herpes simplex virus gD), strain MN, and strain A244 (from CRF01_AE). The ALVAC priming events occurred at weeks 0 and 4, while the protein boost injections were given alongside the ALVAC at weeks 12 and 24 [19].

It is worth noting that both of these components had previously been tested in other trials. ALVAC-HIV, the vector prime, was not as immunogenic as some of the other vectors. AIDSVAX with a bivalent clade B gp120, the protein boost, had also been tested separately with no vector prime, and this was unsuccessful in preventing HIV-1 infection [14].

RV144 trial had a vaccine efficacy of 60% at 12 months and 31% at 3.5 years [17]. Though researchers expected the correlate of reduced risk to be CD8+ T cell response or neutralizing antibodies, the trial results indicated that the strongest correlate of reduced risk was nonneutralizing antibody response to the V1-V2 loop of gp120 [12]. The results also found that high levels of Env-specific IgA antibodies were correlated to infection risk in the vaccinated participants of the RV144 trial. Haynes et al. hypothesized that the high levels of Env-specific IgA antibodies weaken the effects of protective antibodies [20].

Given the unexpected results of RV144, there is renewed interest in the role of antibodies outside the classic role of neutralization, and this is particular focused on antibody-dependent cell-mediated cytotoxicity (ADCC) [12].

### 2.6. Setbacks Following RV144’s Modest Success

Based on the RV144 trial’s modest efficacy, a similar trial in South Africa, HVTN 702, was launched. The vaccine regimen was designed to increase the efficacy and immune response duration of RV144. These modifications from RV144 included changing the clade present in the vaccine due to regional differences, changing the adjuvant in the protein boost from alum to MF49, and changing the timing of the vaccinations from four injections in six months to five injections over twelve months [6].

HVTN 702 was terminated prematurely because the independent data and safety monitoring board found that the vaccine was not effective, with approximately the same number of HIV infections in the participants who received the vaccine as the participants who received the placebo [6].

The rationale behind the study design of HVTN 702 may have led to this product failure in clinical trial. The HVTN 702 clinical trial differed from the RV144 clinical trial in terms of vaccination schedule, clades, subtypes of proteins, lack of tagging of proteins, genes in immunogens, and/or adjuvants. Based on nonhuman primate studies, the alum to MF49 adjuvant change may have contributed to low efficacy in HVTN 702 [19]. MF49 adjuvant was previously utilized to increase neutralizing antibodies and T-cell responses [6] and was tested in another phase I/II clinical trial (HVTN 100). This trial found the regimen to be safe and tolerable, so HVTN 702 proceeded to test its efficacy [19]. More research is needed to understand why HVTN 702 was projected to be more effective than RV144 but showed no efficacy [6].

### 2.7. Broadly Neutralizing Antibodies and the Subsequent Antibody-Mediated Prevention (AMP) Trials

Researchers have attempted to produce immunogens that induce the immune system to synthesize broadly neutralizing antibodies (bNAbs) for several years. BNAbs inhibit the virions from entering the host cells, preventing HIV integration into the genome. Their role is particularly important because bNAbs are able to protect against the strain that the patient has been infected with as well as multiple different immunological strains [21]. Though Env-specific bNAbs are produced in patients with chronic HIV-1 infections, an antibody must undergo extensive somatic mutation with possible insertions or deletions in the immunoglobin heavy and light chains in the germinal center. BNAbs also typically have a third heavy-chain complementarity-determining region (HCDR3) loop, and this feature allows the antibody to combat the Env glycan shield. Some researchers tracked the evolution of the antibody to its development into a bNAb in an effort to understand the generation of bNAb [21]. However, despite all the different versions of HIV envelope glycoproteins studied and synthesized, these glycoproteins or fragments have been unable to elicit a neutralizing response to primary isolates of HIV-1 [7].

The breakthrough that synthesized bNAbs was high throughput single-cell BCR-amplification assays [10]. This was completed by separating HIV-1 Env-reactive memory B cells from antigen-specific B cells, from plasma cells, and from clonal memory B cell cultures [20]. Dozens of new antibodies, including PG6, PG16, and VRC01, have been isolated and characterized based on which target of four highly conserved regions of HIV-1 Env they bind. Scheid classified a set of potent antibodies that mimic CD4 binding as “highly active agonist CD4bs antibodies [10,22]”. This set includes 3BNC117 and VRC01 [10].

To investigate whether these bNAbs could induce a protective immune response in human subjects with HIV-1 infections, two early phase clinical trials were completed. Caskey et al. studied how a passive infusion of 3BNC117 affected the viral load in participants with HIV-1 infections and without HAART treatment [23]. Lynch et al. ran a similar trial with a VRC01 infusion on HAART-treated or HAART-untreated individuals with HIV-1 infections [24]. Both trials testing 3BNC117 and VRC01 indicated that infusion of a bNAb decreased the viral load in participants with HIV-1 infections not on HAART medication [14,23,24]. Since these trials utilized passive immunization, further research is necessary to adapt the bNAb to an active immunization strategy.

Researchers assessed VRC01 further and found that it protected against HIV-1 clades B and C in vitro. Subsequently, they tested VRC01 in two simultaneous proof-of-concept trials, HVTN 703 and 704, starting in 2016. These trials were completed to learn whether this bnAb could prevent HIV-1 acquisition [25].

The results of the antibody-mediated protection (AMP) trials published in 2021, shown in Table 2, indicate that VRC01 was unable to prevent HIV-1 acquisition as compared to the placebo. However, the HIV isolates that were sensitive to VRC01 proved that bnAb does have the potential to prevent HIV-1 acquisition. Corey et al. suggested that multiple potent antibodies could be combined to result in a preventative HIV treatment [25].

### 2.8. Gene Therapy Application to Induce Broadly Neutralizing Antibodies

BNAb as passive immunotherapy would be challenging to administer to a small population with the health infrastructure in place and the cost of biological production. Given the obstacles to administering this therapy, it is clear that an active vaccination eliciting bNAbs would be more effective as a prophylactic vaccine. A concept to circumvent this issue is vectored immunoprophylaxis, where an adeno-associated vector and the bnAb genes are injected into the muscle. Researchers used this gene therapy concept and conducted a phase 1 trial (IAVI A003/CHOP HVDDT 001) with rAAV vector coding for PG9 antibody, shown in Table 2. The results indicated that more research needs to be completed to increase the antibody expression because the PG9 antibody level was detected indirectly. Overall, it was safe and well-tolerated in the 16 participants tested [26].

### 2.9. Mosaic Vaccine Design, APPROACH, and HVTN 705

Given HIV-1’s vast genetic diversity and several strains, some researchers reconstructed global HIV-1 sequences in silico to generate mosaic immunogens [10]. These immunogens have the maximum of potential T cell epitopes and, if administered as a vaccine, induce broader cellular and humoral immune responses as compared to wild-type of consensus HIV-1 antigens [27]. In theory, mosaic antigens could generate a global HIV-1 vaccine [28]. A phase I clinical trial testing mosaic Env and Gag-pol antigen set in adenovirus serotype 26 proved that the vaccine induced strong Env-specific immune responses in the bloodstream and colorectal mucosa [28].

APPROACH, a phase I/II clinical trial, investigated the safety and effects of two different mosaic vaccines in Ad26 or MVA vectors followed by a protein boost of Env gp140 (Table 2). At the end of the study, Env-specific binding antibody responses were measured for each experimental group. Though both vectors were safe and well-tolerated, Ad26 had the strongest immunogenicity. Barouch et al. found that Env-specific binding antibody responses, antibody-dependent cellular phagocytosis responses, and T-cell responses were present in 100%, 80%, and 83% of participants. This research group also administered a similar vaccine regimen in rhesus monkeys and found that it provided a 66% protection against SHIV-SF16P3 infection in six virus challenges [28]. While this is an exciting development, it is important to note that the virus challenges consisted of one strain and the mosaic vaccine may not be able to protect against different strains [27]. 

Building on the positive phase I/II results of the Ad26 mosaic vaccine, a phase II clinical trial, HVTN 705/Imbokodo, evaluated the efficacy and was expected to be completed in 2022 [28]. Unfortunately, this study was terminated in 2021 after the primary endpoint results showed that the vaccine did not confer any statistically significant efficacy. This study proved that the necessary immune response to confer protection against HIV is greater than the immune response to confer protection against other viruses such as COVID-19 [29]. The Ad26 vector was successfully utilized to manufacture Ad26.COV2.S, a recombinant vaccine to protect against COVID-19 [30].

### 2.10. HIVconsv Vaccine

Some research groups believe that a rationally designed HIVconsv immunogen is the key to an effective HIV vaccine. In theory, this immunogen causes T-cells to bind conserved regions of HIV-1 proteins and is designed with alternating clade consensus sequences between each conserved sequence. In humans, a vaccine of HIVconsv inserted into DNA, simian adenovirus, and poxvirus MVA vectors induce broadly specific T cell responses [8].

A phase I clinical trial, known as HIV-CORE 004, evaluated pSG2.HIVcons plasmid DNA, MVA.HIVconsv and Ad35-GRIN. Ad35-GRIN contained HIV-1 clade A Gag, reverse transcriptase, integrase, and Nef fusion protein (GRIN). All participants had HIVconsv-specific T cell responses. In vitro, vaccine-induced T-cells inhibited replication of the majority of viruses tested from HIV-1 clades A, B, C, and D [8].

A second generation tHIVconsvX, based on this concept, will be studied in HIV-CORE 0052, a phase I clinical trial. This is designed to test the safety and immunogenicity of the conserved mosaic HIV-1 vaccine [31].

### 2.11. mRNA-Based Vaccine Technology for HIV

In an attempt to produce an effective HIV-1 vaccine, researchers are developing messenger ribonucleic-acid (mRNA)-based vaccines. mRNA vaccines have been developed for Zika, influenza [32], and 2 vaccines for COVID-19 virus, BNT162b2 (Pfizer-BioNTech, New York, NY, USA) [33] and mRNA-1273 (Moderna, Cambridge, MA, USA) [34], and this is in part due to the lack of infectious risk, ease of manufacturing, and flexibility of immunogens associated with this type of vaccine.

Given the biological challenges of HIV-1, as discussed previously, this strategy to vaccination will likely be more complicated than other strategies. Mu et al. hypothesized that this scenario for a possible effective HIV-1 mRNA vaccine involves a sequential immunization of mRNAs encoding Env-based immunogens that will produce germline precursors that develop into bNAb in the germinal centers [21].

In phase 1 clinical trial of an mRNA-based HIV vaccine, IAVI G001, researchers administered participants with two doses of eOD-GT8 60mer vaccine or placebo [35]. The eOD aspect of this nanoparticle-mRNA vaccine is an engineered outer domain of the Env gp120 binding CD4. This eOD design of the vaccine targets germline B cells and helps mature them into bNAB [32]. The initial results presented at the HIV Research and Prevention virtual conference indicated that 97% of participants who received the eOD-GT8 60mer vaccine developed VRC01-class IgG B cells. These B cells are considered to be the progenitor to the VRC01 class of bNAbs. While this is an exciting development, it is essential to consider that the results have not been peer reviewed and that VRC01 antibodies must mutate to become bNAbs [35]. A similar phase 1 clinical trial, IAVI G002, is slated to begin in September 2021 [36].

### 2.12. Future Directions

Though there have been significant achievements in preventing HIV-1 infections in at-risk populations, it does not replace the necessity for the efficacious prophylactic vaccine. This has become apparent now more than ever because of the pandemic of COVID-19. This pandemic illustrated the significant gaps in healthcare and its accessibility to populations at risk for developing HIV-1 infections. People at risk for developing HIV-1 infections and people with HIV-1 infections have been negatively impacted in terms of HIV testing, treatment access, availability of preexposure prophylaxis, and treatment of opportunistic infections [37]. The success of developing a vaccine to protect against COVID-19 in a relatively short time frame involved collaborations, and it has renewed interest in the development of a prophylactic HIV-1 vaccine [38].

## 3. Conclusions

More research, funding, and clinical trials are necessary to eradicate the HIV-1 epidemic. Though researchers have been developing a vaccine for thirty years, the mosaic antigens, broadly neutralizing antibodies, and gene therapy application to induce broadly neutralizing antibodies show significant advancement and may potentially provide us with an HIV-1 vaccine in the future.

## Figures and Tables

**Table 2:** Recent and ongoing HIV-1 clinical trials.

Name	Year Started and Country/Continent	Phase	Molecular Basis of Vaccine	Efficacy/Response or Completion Date	Relevant Comments	NCT Number or Author
HVTN 702	2016, South Africa	2b/3	ALVAC-HIV and subtype C gp120/MF59	No efficacy		NCT02968849
Antibody-Mediated Protection, HVTN 703	2016, sub-Saharan Africa	2b	VRC01 broadly neutralizing monoclonal antibody infusion	Did not prevent HIV-1 acquisition	Similar to HVTN 704 but clinical participants were women	NCT02568215
Antibody-Mediated Protection, HVTN 704	2016, Brazil, Peru, Switzerland, USA	2b	VRC01 broadly neutralizing monoclonal antibody infusion	Did not prevent HIV-1 acquisition	Similar to HVTN 703, but clinical participants were men who have sex with men and transgender women	NCT02716675
IAVI A003/CHOP HVDDT 001	2014, United Kingdom	1	rAAV1-PG9DP	Safe and tolerable, but antibody levels not detected in all participants		NCT01937455
APPROACH	2015, east Africa, South Africa, Thailand, and USA	½	Ad26.Mos.HIV and Clade C gp140 or MVA mosaic vaccine with gp140	Safe and tolerable, efficacy will be assessed with HVTN 705		NCT02315703
Imbokodo, HVTN 705	2017, sub-Saharan Affrica	2b	Ad26.Mos4.HIV and adjuvanted clade C gp140 and Mosaic gp140 protein	Completion date in 2022		NCT03060629
HVTN 706	2019, Europe, North, and South America	3	Ad26.Mos4.HIV and adjuvanted clade C gp140 and Mosaic gp140 protein	Completion date in 2022		
CR108152	2016, USA and Rwanda	½	Ad26.Mos.HIV or Ad26.Mos4.HIV and clade C gp140 plus adjuvant	Completion date in 2023		NCT02788045
HIV-CORE 004	2014, Kenya	½	pSG2.HIVconsv DNA, MVA.HIVconsv and Ad35-GRIN	Safe and tolerable, all participants had HIVcons specific T cell responses		NCT02099994
HIV-CORE 0052	2021, United Kingdom	1	ChAdOx1.tHIVconsv1, MVA.tHIVconsv3 (M3), or MVA.tHIVconsv4 (M4)	Completion date in 2022		NCT04586673
PrepVacc	2020, Mozambique; South Africa; Tanzania; and Uganda	2b	DNA-HIV-PT123 and AIDSVAX or 2 injections of CN54gp140 + MPLAL and MVA	Completion date in 2023	All participants on PREP	NCT04066881
IAVI G001	2018, USA	1	eOD-GT8 60mer + AS01B/DPBS sucrose/IM	Results have not been published in peer reviewed journal		NCT03547245
IAVI G002	Estimated start date 2021, USA	1	Core-g28v2 60mer mRNA and eOD-GT8 60mer mRNA Vaccine	Completion date in 2023		NCT05001373

## Data Availability

Not applicable.
